# Miniscrew-supported distal jet versus conventional distal jet appliance: A pilot study

**DOI:** 10.4317/jced.55780

**Published:** 2019-07-01

**Authors:** Michele Cassetta, Giulia Brandetti, Federica Altieri

**Affiliations:** 1 DDS, PhD (Associate Professor) Department of Oral and Maxillofacial Sciences, “Sapienza” University of Rome, Italy, School of Dentistry; 2 MS (Research Assistant) Department of Oral and Maxillofacial Sciences, “Sapienza” University of Rome, Italy, School of Dentistry; 3 DDS, PhD (Research Assistant) Department of Oral and Maxillofacial Sciences, “Sapienza” University of Rome, Italy, School of Dentistry

## Abstract

**Background:**

Maxillary molar distalization is the most frequently used nonextraction treatment in the correction of Class II malocclusion. The use of traditional intra-oral devices shows unreliable results. Nowadays the use of miniscrew-supported appliances helps prevent anchorage loss. The aim of this pilot study is to compare the amount of upper first molar distalization and the dentoalveolar side effects using traditional distal jet appliance and miniscrew-supported distal jet appliance.

**Material and Methods:**

20 patients were randomly assigned to receive a treatment with miniscrew-supported distal jet appliance (Group A) or with traditional distal jet appliance (Group B). To ensure a safe and minimally invasive miniscrew insertion a surgical guide was used. Digital models and lateral cephalograms were obtained and analyzed before orthodontic treatment and at 6-month follow-up. Intergroup differences were determined using T- test. The significance was set at *p* ≤0.05. The intra-operator reliability was evaluated using a 2 sample T-test. The difference was not statistically significant (*P* ≤0.05 ), demonstrating an intra-operator reliability.

**Results:**

In Group A, a greater maxillary first molar distalization was recorded (*P*=0.002). Considering the dentoalveolar side effects, in Group A, a spontaneous distalization of the first premolars and a retroclination of central incisors were determined. In Group B, the first premolars tipped mesially with a proclination of the maxillary central incisors.

**Conclusions:**

Miniscrew-supported distal jet appliance achieved a greater first molar distalization at 6-month follow-up and did not cause dento-alveolar side effects, such as the mesial drift of the premolars and the incisors.

** Key words:**Molar distalization, distal jet appliance, skeletal anchorage, miniscrew, 3D printed surgical guide.

## Introduction

Maxillary molar distalization is a nonextraction procedure normally used to gain space in the upper arch and to correct a Class II dental relationship (when there is a mesial migration of maxillary posterior teeth). Traditionally extraoral appliance, the headgear, is used for maxillary molar distalization (1). However, headgear needs patient compliance and it is esthetically unacceptable. Intraoral devices are then developed (eg. pendulum appliance, distal jet appliance) (2-4); these appliances do not require patient cooperation but cause, as a side effect, the mesial drift of the premolars and the incisors, namely anchorage loss (2-4). To prevent anchorage loss, intraoral distalization appliance supported by additional miniscrew anchorage can be used (5,6).

The aim of the present study was to compare the amount of upper first molar distalization and the dentoalveolar side effects, obtained in six months, using a traditional distal jet appliance and a miniscrew-supported distal jet appliance. The results were analyzed at 6 months from the start of therapy. This endpoint was determined to avoid that different pattern of malocclusion influenced the results, but in all patients the therapy continued until a first molar relationship was achieved.

It was hypothesized that miniscrew-supported distal jet appliances achieve a greater first molar distalization in six months and do not cause dentoalveolar side effects as the premolar and incisor mesial drift.

## Material and Methods

This prospective case-control study was conducted at the Department of Orthodontics of “Sapienza”, University of Rome between June 2017 and April 2018. The STROBE (Strengthening the Reporting of Observational Studies in Epidemiology) guidelines for prospective studies were followed. The clinical investigation was conducted in accordance with the ethical principles of the World Medical Association Declaration of Helsinki. The parents or guardians were informed of the content, risks, and benefits of the study and a written consent was obtained. The investigation was independently reviewed and approved by the local ethics committee ( No. 3802 ).

One hundred and fifty patients were examined within two months. The first patient was enrolled on 1 June 2017 and the last patient was enrolled on 31 July 2017. All treatments were completed by April 2018.

Eligibility criteria were as follows.

• Caucasian children over 12 years of age.

• No previous orthodontic treatment.

• No systemic syndrome involved;

• Good oral hygiene;

• Bilateral II class malocclusion with a mesial migration of maxillary posterior teeth;

• Lack of sufficient space for proper eruption of one or both maxillary canines;

• Presence of upper second molars.

Only 20 patients met the eligibility criteria. The patients were randomly assigned to receive a treatment with miniscrew-supported distal jet appliance (Group A) or with traditional distal jet appliance (Group B). To obtain the treatment allocation, a randomization sequence was created using CLINSTAT (Martin Bland, York, United Kingdom) statistical software.

**Figure 1:**
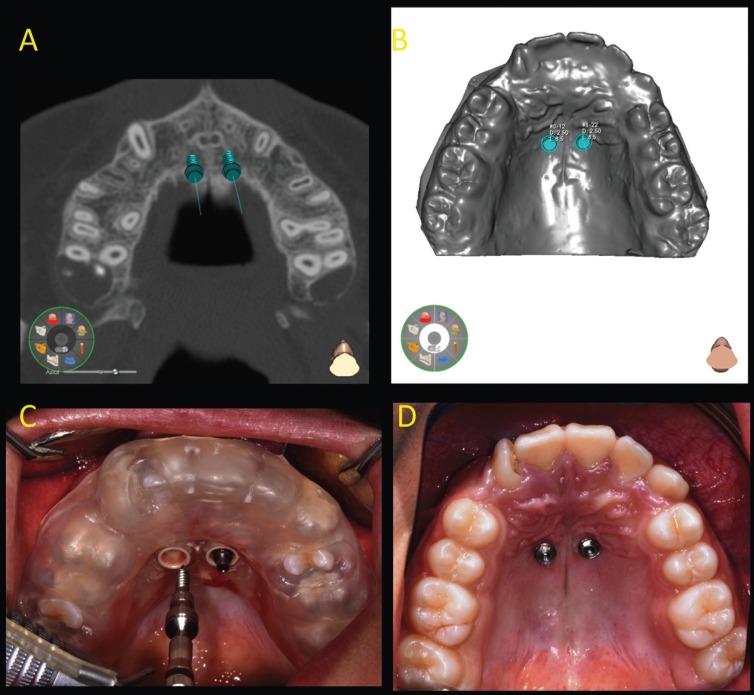
A) Virtual miniscrew placement using the three-dimensional computer planning software. B) Using the software, it is also possible to evaluate the position of miniscrews on the virtual dental cast. C) The surgical guide, teeth-supported, held in place. D) Self-drilling miniscrews placement in the anterior region of palate.

**Figure 2:**
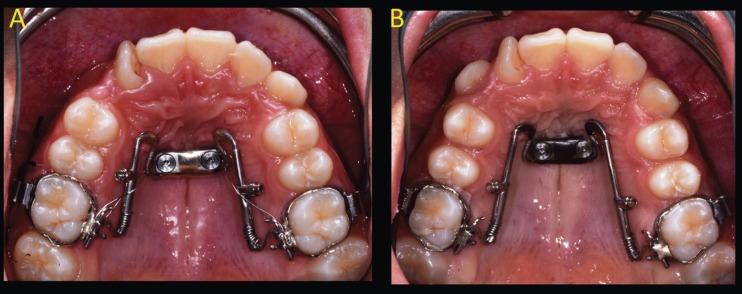
A) Miniscrew-supported distal jet appliance at the beginning of treatment. B) 6- month follow-up: occlusal view shows molar distalization and a spontaneous second premolar dental drifting.

**Figure 3:**
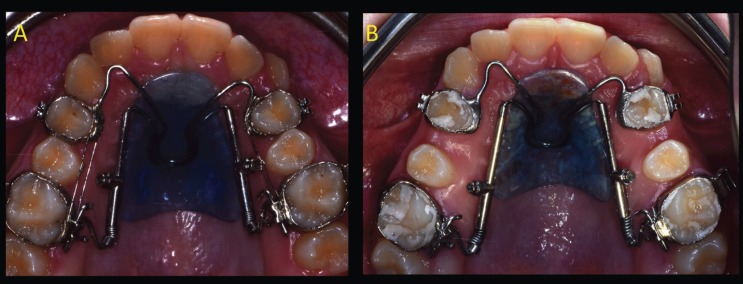
A) Occlusal view immediately after distal jet placement. B) Occlusal view at 6-month follow-up.

Traditional distal jet appliance is an intraoral palatal device, which exerts its effects with the compression of Nichel-Titanium (Ni-Ti) coil spring between the banded upper first molars, banded upper first premolars and the Nance button (2-4). The miniscrew-supported distal jet appliance has 2 miniscrews inserted in paramedian position of palatal vault and the coil spring is compressed between the banded upper first molars and the miniscrews. To ensure a minimally invasive miniscrew insertion a surgical guide was created using a 3D printer (7). The appropriate miniscrew insertion sites were planned on 3D images created by the fusion of CBCT and dental digital model images (Fig. 1 A-B). The length and diameter of titanium self-drilling miniscrews used (Vector Ortho, Whitek, Lodi, Italy) was predetermined in the planning phase (Fig. 1 B) (7). Before miniscrews insertion, the patient’s mouth was rendered aseptic by rinsing with a 0.2% chlorhexidine solution. Local anaesthesia was performed with 2% carbocain containing adrenaline in the ratio of 1:100,000. The surgical guide was held in place by a tooth-borne shape to ensure stable retention and was used for miniscrew insertion. (Figure 1 C-D). In both groups a superelastic Ni-Ti coil spring with a force of 250 N was compressed to achieve the force needed for molar distalization. The coil spring was reactivated every month until the sixth month (Figs. 2,3). Initial pre-orthodontic treatment ( T0 ) and at 6-month follow-up ( T1 ) digital models and lateral cephalograms were obtained and analyzed to evaluate the amount of upper first molar distalization and the dentoalveolar side effects . On dental cast the following measurements were registred according to the method of Hass and Cisneros (8), Hoggan and Sadowsky (9) and Kinzinger *et al.* (10):

• the distance from the distal point of contact of the right lateral incisor to the mesial point of contact of the right first molar (UR2-UR6 ) and the distal point of contact of the left lateral incisor to the mesial point of contact of the left first molar (UL2-UL6);

• the transverse width change of the maxillary first molars measured from the mesiobuccal cusp tip of the right maxillary molar to the mesiobuccal cusp tip of the left maxillary molar ( mesiobuccal cusp tips UR6-UL6), from the distobuccal cusp tip of the right maxillary molar to the distobuccal cusp tip of the left maxillary molar (distobuccal cusp tips UR6-UL6) and from the lowest point of central fossa of the right maxillary molar to the lowest point of central fossa of the left maxillary molar (central fossa UR6-UL6);

• the transverse width change of the maxillary second premolar measured from the central fossa of the right second bicuspid to the central fossa of the left second bicuspid ( central fossa UR5-UL5);

• the distance from tangent to inferior border of palatal rugae, perpendicular to midpalatal raphe, to the perpendicular to midpalatal raphe, at mesiobuccal cusp tip both of right maxillary first molar (line AR) and of left maxillary first molar (line AL) and the distance from tangent to inferior border of palatal rugae, perpendicular to midpalatal raphe to the perpendicular to midpalatal raphe, at mesiolingual cusp tip both of right maxillary first molar (line BR) and of left maxillary first molar (line BL);

• the angle between a line running through the mesiobuccal and mesiolingual cusps of the first maxillary molars and the midpalatal raphe ( tooth UR6 rotation, tooth UL6 rotation).

On pre- ( T0 ) and post-treatment ( T1 ) lateral cephalograms, the cephalometric analysis was performed using Oris Ceph software (Oris Ceph, Elite Computer, Vimodrone, Milano, Italy).

In order to reduce the bias in the selection of subjects, considering that the sample size was too small to represent the entire spectrum of subjects in the target population, the results were analyzed at 6 months from the start of therapy. All patients were treated by a single operator, an expert orthodontist and oral surgeon (MC). The confounding factors were reduced by excluding the patients with upper second molars unerupted from the study.

-Statistical analysis

A database was created using Excel (Microsoft, Redmond, WA, USA), with appropriate checks to identify errors. Data were evaluated using the statistical analysis software SPSS® v. 17.0 (IBM Corporation, Armonk, NY). For the statistical analysis, descriptive statistics including mean values and standard deviation were used to evaluate the amount of upper first molar distalization and dentoalveolar side effects, at 6-month follow-up. T- test was used to analyze intergroup differences. The significance was set at *p* ≤0.05. To calculate the intra-operator reliability, all measurement were repeated by the same examiner (MC) after 4 weeks and the differences evaluated using a 2 sample T-test. The difference was not statistically significant ( *P* ≤0.05 ), demonstrating an intra-operator reliability ( Table 1).

**Table 1:**
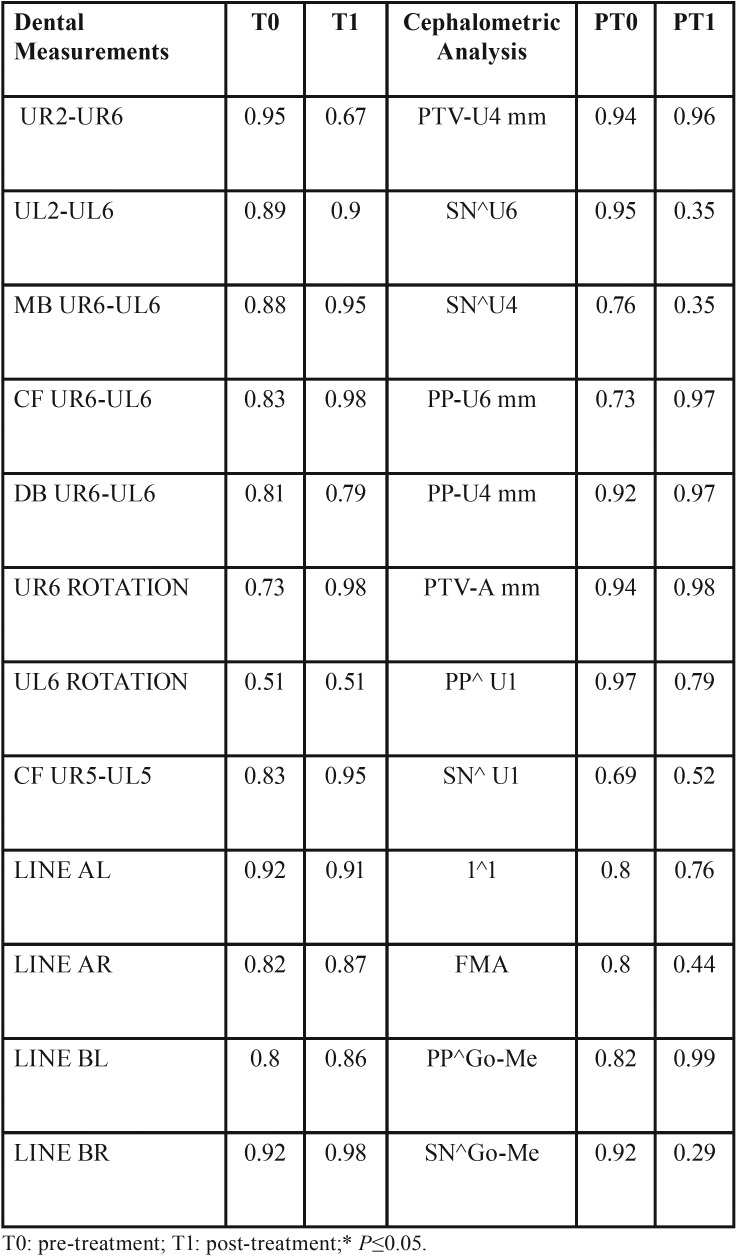
2 sample T-test to calculate the intra-operator reliability.

## Results

The study sample comprised 20 subjects (9 males and 11 females). Ten patients ( mean age 13.1 years) were randomly assigned to receive a treatment with miniscrew-supported distal jet appliance (Group A) and ten (mean age 12.3years) were treated with a traditional distal jet appliance (Group B). Baseline characteristics including age, sex, II class malocclusion with a mesial migration of maxillary posterior teeth were similar in the two groups. All the patients included in the study completed the 6-month follow-up. No data was missed for each variable of interest. All screws were stable at the time of insertion, at six-month follow-up, and at the end of treatment.

Considering the differences between the dental cast measurements at T0 and T1 (∆T1-T0) recorded in both groups, the distance from the distal point of contact of the lateral incisor to the mesial point of contact of the first molar increased in the Group A on average of 5 mm in the first quadrant and of 4.2 mm in the second quadrant. In the Group B the distance from the distal point of contact of the lateral incisor to the mesial point of contact of the first molar increased of 3.5 mm in the first quadrant and of 3.4 mm in the second quadrant with a statistically significant difference between groups for UR2-distal-UR6 mesial value (*P*=0.03). The transverse widths of the dental arch increased between the mesiobuccal cusps, the central fossae, and between the distobuccal cusps in both groups ( Table 2); this indicates an expansion of dental arch. Evaluating the molar rotation, in the Group B the permanent first molars recorded a greater mesial rotation (UR6 rotation: -4.2°; UL6 rotation:-9.8°) compared to Group A ( Table 2). The upper molars moved in distal direction of 3.6 mm and 3.8 mm (lines BL;BR) in the Group A, whereas in the Group B the distalization was marginal (line BL: 0.3 mm; line BR: 0.6 mm).

**Table 2:**
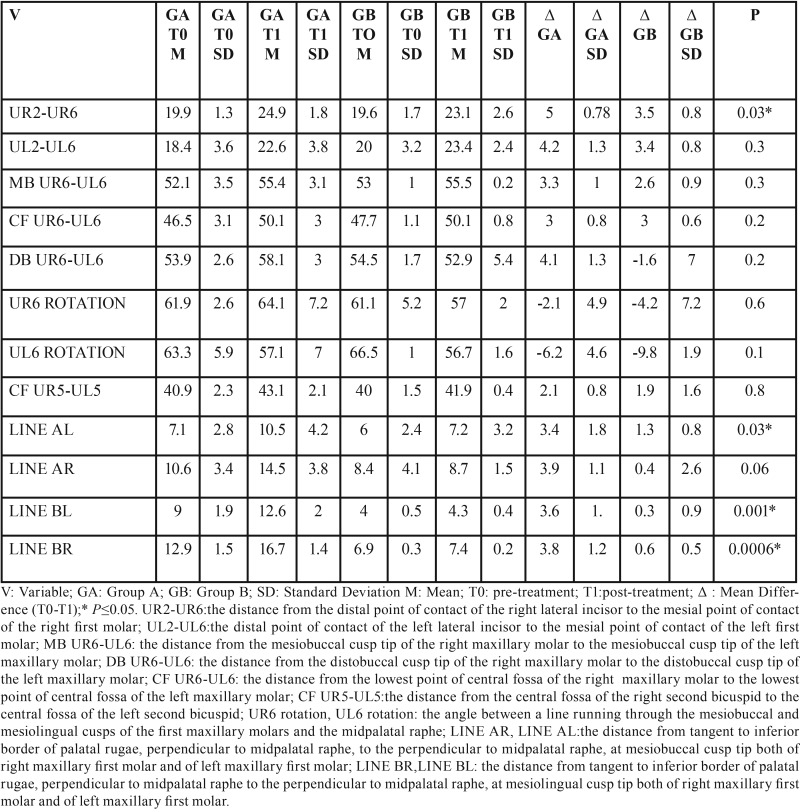
Dental cast linear and angular measurements.

Cephalometric data and results are shown in  Table 3. The mean first maxillary molar distalization (PTV-U6) was 5.3 mm ±2.1 mm and 0.9 mm± 0.9 mm in groups A and B, respectively. The difference between the two groups was statistically significant (*P*=0.002). In both groups the first upper molar distal tipping in relation to the anterior cranial base (SN-U6) was close to zero ( Table 3). It was interesting to note that in the Group B the first premolars tipped mesially by 6° in relation to the anterior cranial base (SN^U4), instead in Group A the first premolars were distalized by- 4.3 mm (PTV-U4), extruded by 0.5 mm (PP-U4) and tipped distally by 7.7° (SN^U4). During the molar distalization phase of treatment, in Group B the mean proclination of the maxillary central incisors amounted to 3.6° and 4° in relation to the palatal plane (PP^U1) and to the anterior cranial base (SN^U1) whereas in the Group A there was a retroclination of central incisors ( PP^U1= -2.1°; SN^U1= -3.7° ). Vertical and sagittal dimensions remained virtually unchanged in both groups ( Table 3).

**Table 3:**
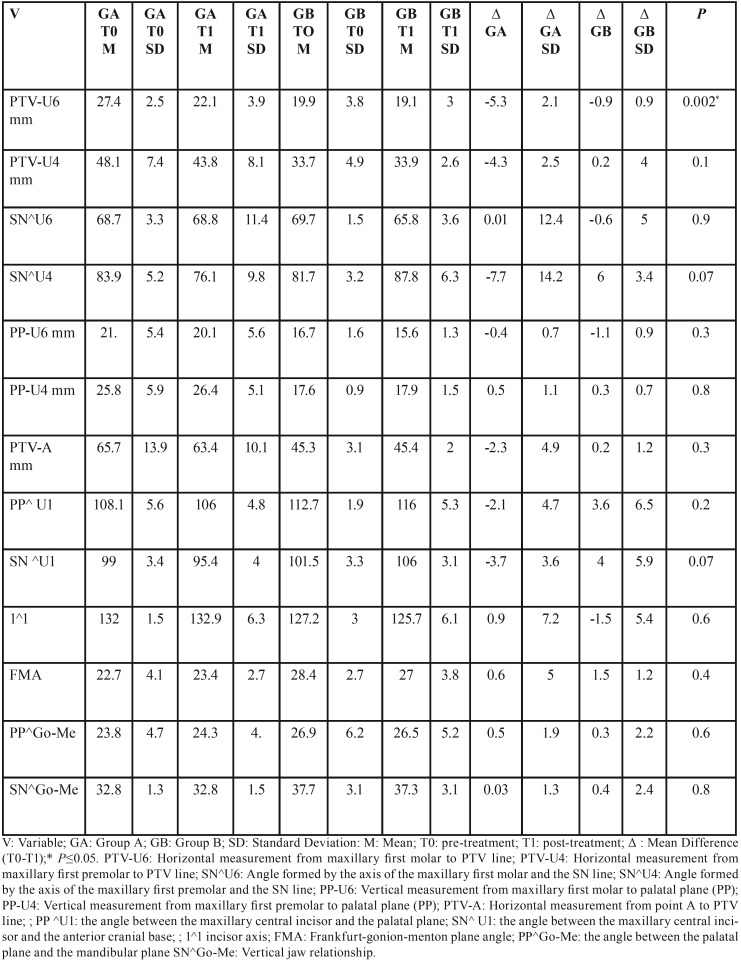
Cephalometric data.

## Discussion

In the present study, two maxillary molar distalization systems, supported and not-supported by intraosseous miniscrew, were compared and the clinical effects evaluated at 6-month follow-up.

The results confirmed the hypothesis: the miniscrew-supported distal jet appliance achieved a greater first molar distalization at 6-month follow-up and did not cause dento-alveolar side effects, as the mesial drift of the premolars and the incisors.

Considering the limitations of the present study, the possible random error arising from the small sample size must be evaluated. However, the present was a pilot study and its purpose was to determine the sample size of a subsequent randomized controlled trial (RCT). Therefore, the present results must be interpreted with caution and subsequently confirmed. Regarding the possible systematic error and therefore the possible sources of bias, a limitation of the present study was represented by the lack of blinding.

In the actual study the dental cast measurements showed that the distance from the distal point of contact of the lateral incisor to the mesial point of contact of the first molar (called supporting zone) had increased in both the maxillary molar distalization systems, supported (Group A ) and not supported ( Group B ) by intraosseous miniscrew. These results are in according to Kinzinger *et al.* that investigated the suitability of the skeletonized distal jet appliance with two paramedian miniscrews for additional anchorage (10). The same Authors (10) found an increase of supporting zone after molar distalization. However, the measurements of supporting zone can be influenced by the variation of the lateral incisor position during the canine eruption (ugly duckling stage). Accordingly, in the present study both digital dental cast and lateral cephalogram were evaluated, determining that the increase of the supporting zone was due to a molar distalization using an intraosseous miniscrew-supported appliance ( Group A ) and due to a premolar and incisal mesial inclination using a not-supported appliance ( Group B ). In the present study mesial inward and distal outward rotations of the maxillary molars were also observed. This effect, as observed by other Authors (10), finds a biomechanical explanation: when the force is applied palatally from the center of resistance of the molars, a rotation of these teeth occurs. Evaluating the cephalometric analysis data, the maxillary first molars were distalized 5.3 mm on average (PTV-U6) in the Group A. In the same group, dental cast measurements showed an average increase of distance from palatal rugae to the mesiobuccal cups and mesiopalatal cusps, confirming the distal movement of upper first molars (lines AR, AL, BR, BL). Instead, in the Group B a maxillary incisors proclination (SN^U1: 4.0°; PP^U1:3.6°) rather than an upper molar distalization was observed. In both groups a bodily movement was recorded instead of a distal tipping of maxillary molars; this result can be explained by the presence of second maxillary molars in the arch. As stated by Kinzinger *et al.* (10) the distal tipping of first upper molars is relatively greater when the second molars are only germinating because they have the same effect as a lever pivot point on the upper first molar to be distalized. When the root of permanent second molar is developing and is erupting, the point of contact between the two molars moves coronally and the tendency for the first molar to tip decreases (11). A significant spontaneous first premolar distalization was observed in the Group A (PTV-4: - 4.3 mm), while in the Group B a mesial tipping of the premolar crowns was observed rather than a real mesialization (SN^U4: 6.°; PTV-4:. 0.2 mm); this result may be explained by the fact that in the Group A first premolars are not bonded and therefore pulled by transeptal fibers in a more distal position. These results are in according to Cozzani *et al.* (12) that compare a bone anchored appliance to the traditional tooth-supported appliance. Also Kinzinger *et al.* (10) find a spontaneous distal drifting only of the second premolar which was not part of the anchorage setup and a mesial drifting of the first premolar, included in the anchorage setup. Regarding the premolar extrusion, a slight extrusion was observed in both groups, greater in Group A compared to Group B: this is explained by the fact that in Group A, premolars are not bonded. These findings were similar to those of Cozzani *et al.* (12), which observed an average extrusion of 1.1 mm in the distal screw group and of 3.5 mm in the control group, over a period of 10 months. The results of this pilot study showed that traditional tooth-supported distal jet appliance produces a mesial drifting of the premolars and a labial tipping of the maxillary incisors. These results are in agreement with other studies (4,11) which state that the anchorage support of traditional distal- jet appliance cannot completely resist the mesial reciprocal force of this type of molar distalization appliance. Studies on bone anchored appliances (10,12) showed a more efficient control in anterior anchorage loss and a decrease in treatment time. Based on a recent systematic review results finalized to evaluate the quantitative effects of miniscrew-supported appliances for maxillary molar distalization in Class II malocclusion, it can be stated that: miniscrew anchorage not only causes distal movement of premolars but also causes a significant distal movement of incisors, preventing upper incisors from flaring (13). The palatal tipping of the maxillary incisors is described with the use of different bone-anchored appliances for maxillary molar distalization (13). The only study that evaluates the position of the incisors, using a skeletonized distal jet, reports a minimal upper central incisors proclination (10). These results disagree with the present study, but the different appliance design (presence/absence of occlusal dental rest on the first premolars) could explain this difference. This aspect should be further investigated to determine the real undesirable effects of miniscrew-supported distal jet. To date, however, there are no randomized controlled prospective studies in the literature, and this pilot study is preparatory to a subsequent RCT.

Miniscrew-supported distal jet appliance can be used safely for first upper molar distalization. In a short treatment time (6 months), an effective molar distalization without relevant dentoalveolar effects is possible without an anchorage loss. A palatal inclination of central incisors and the distally drifting of first premolars can occur when miniscrew-supported distal jet appliance is used.
